# Supplementary material for: Heteroleptic Complexes of Ruthenium Nitrosyl with Pyridine and Bypiridine—Synthesis and Photoisomerization
Source: Molecules. 2024 Aug 26;29(17):4039. doi: 10.3390/molecules29174039 (PMC11397342; doi:10.3390/molecules29174039)
Supplement: Supplementary file 1 [file molecules-29-04039-s001.zip › molecules-3143303-supplementary.pdf]

SUPPLEMENTARY INFORMATION

To the article of **Brovko A.O.[1], Pischur D.P.[1], Kuratieva N.V.[1], Kostin G.A.[1]**

Heteroleptic complexes of ruthenium nitrosyl with pyridine and bypiridine – synthesis and photoisomerization

Nikolaev Institute of Inorganic Chemistry SB RAS, Novosibirsk, Russia

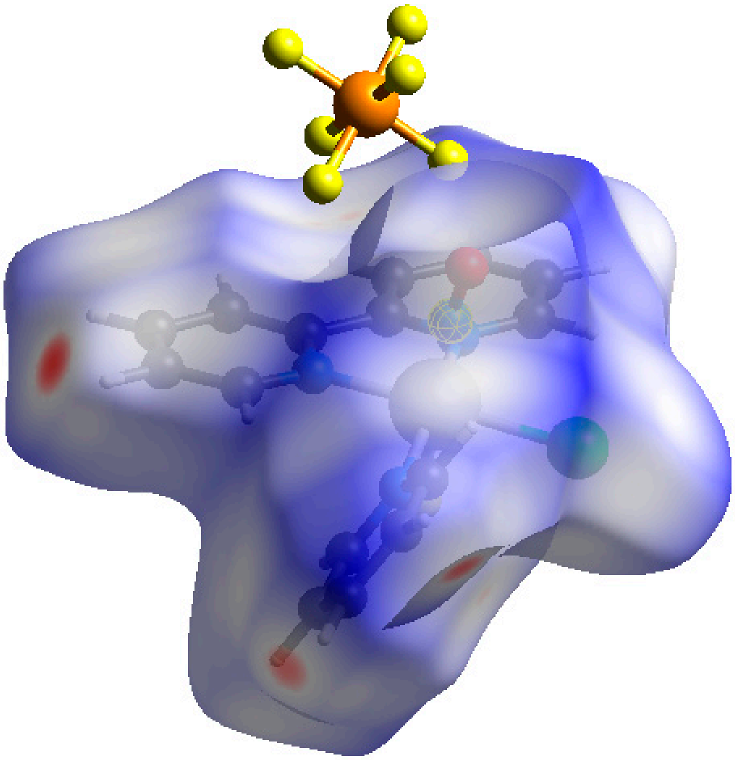

Figure. S1 Hirshfeld surface for the cation **[RuNO(Py)(Bpy)ClF]PF$_6$**

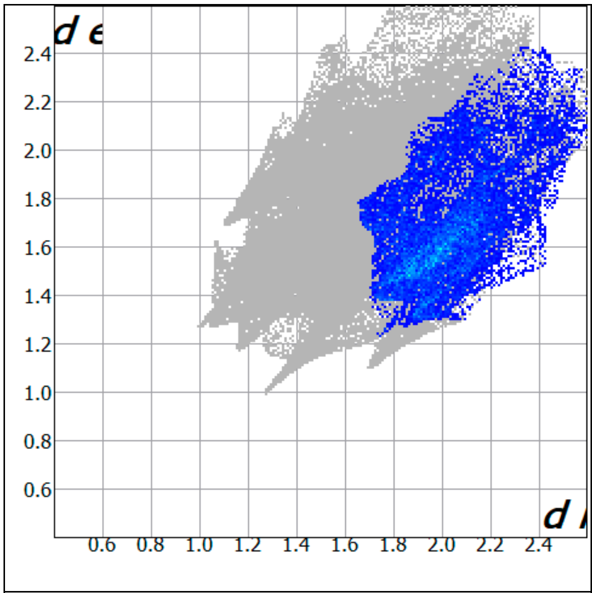

C to all (18.3 %)

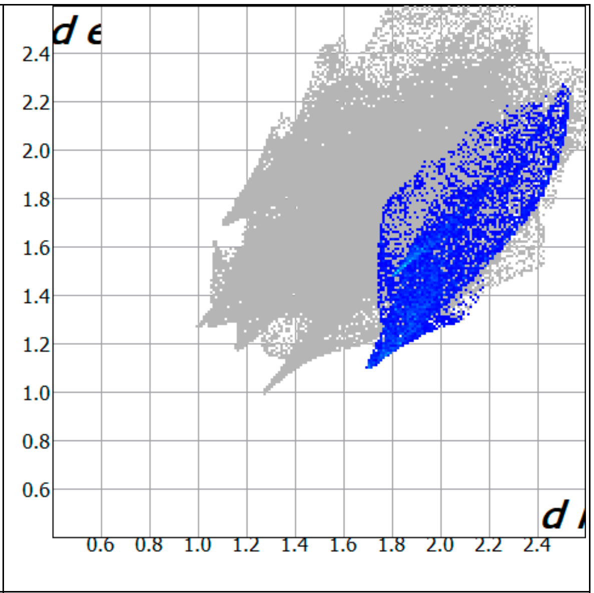

Cl to all (7.5 %)

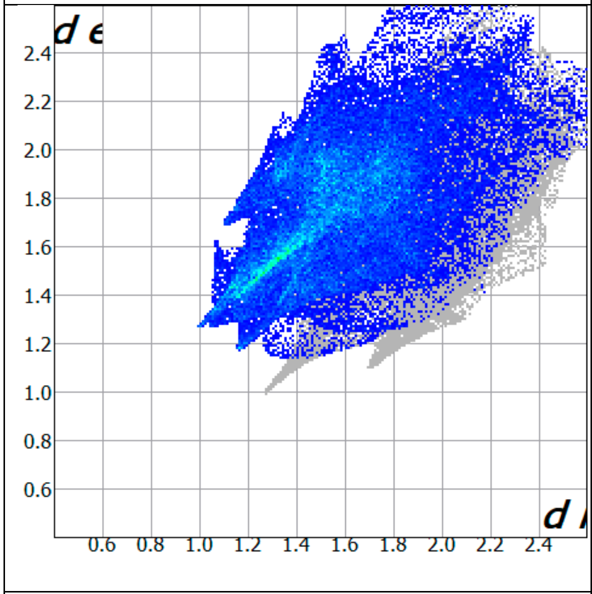

H to all (58.9 %)

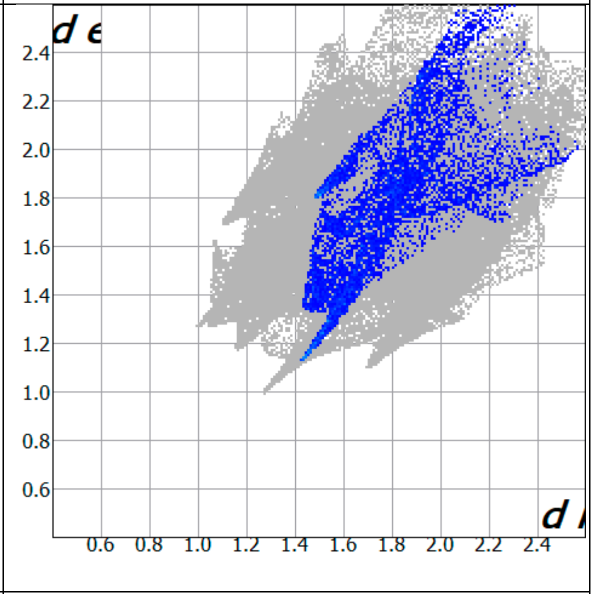

O to all (7.2 %)

Figure S2. Fingerprints of Hirshfeld surface demonstrating short contact of different kind of cation atoms in **[RuNO(Py)(Bpy)ClF]PF$_6$**

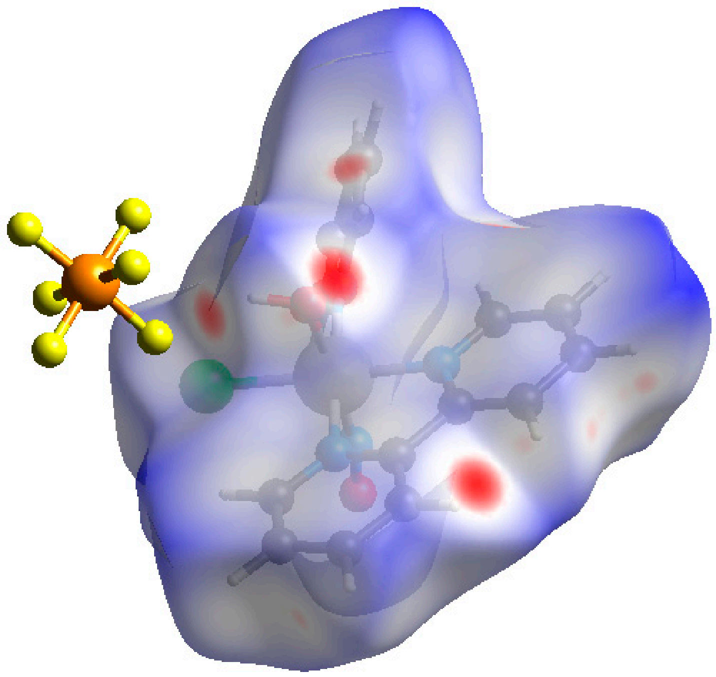

Figure. S3 Hirshfeld surface for the cation **[RuNO(Py)(Bpy)ClOH]PF$_6$**

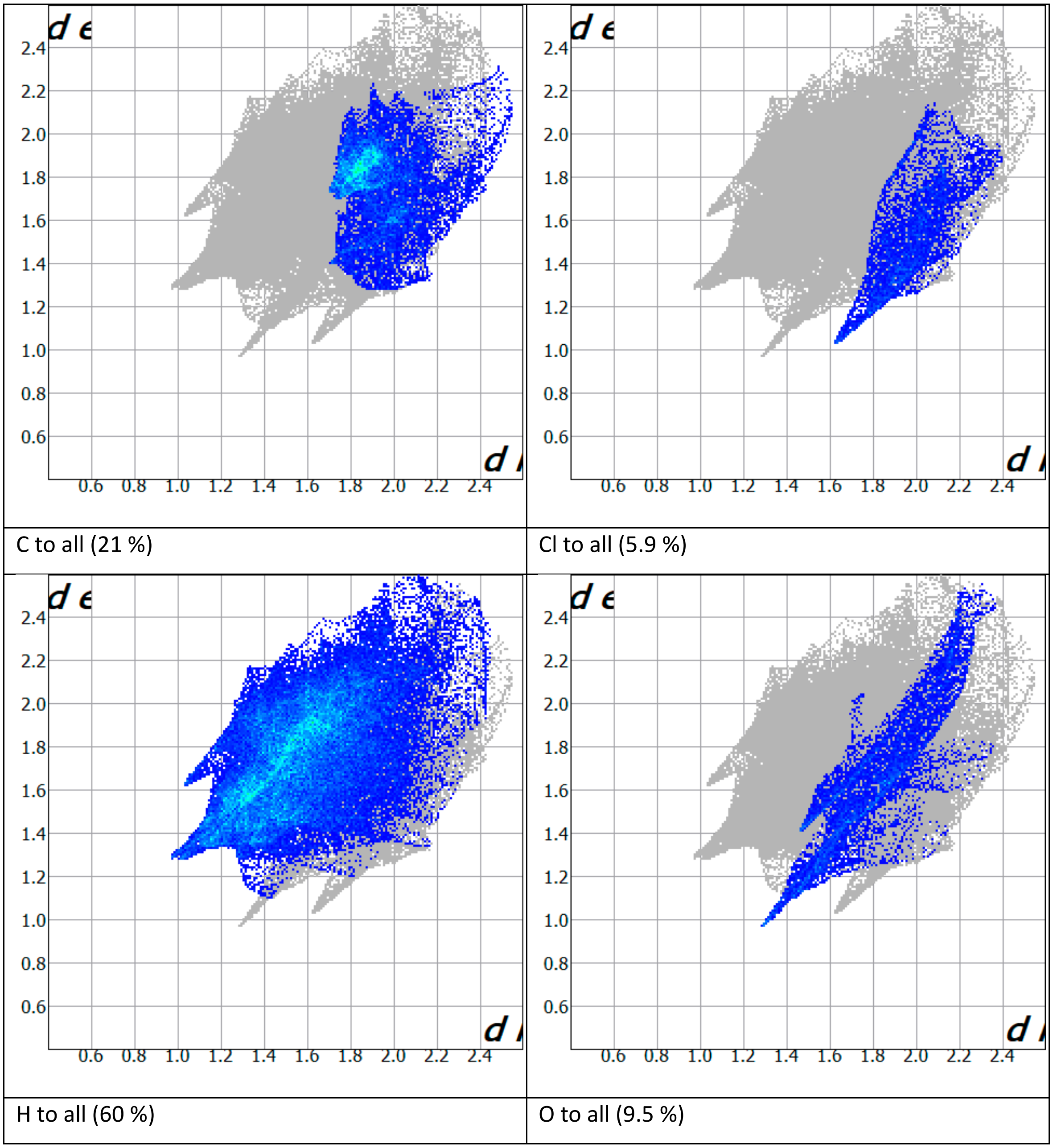

Figure S4. Fingerprints of Hirshfeld surface demonstrating short contact of different kind of cation atoms in **[RuNO(Py)(Bpy)ClOH]PF$_6$**

Table S1. The picture and the main atomic contributions (%) in the molecular orbitals in investigated complexes

Orbital number	$[RuNO(Bpy)PyClF]^{+}$	$[RuNO(Bpy)PyClOH]^{+}$
91	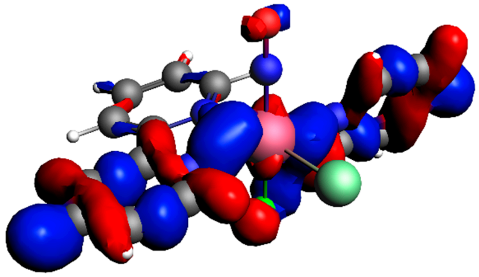 F(9), Ru (10), organic ligands (54)	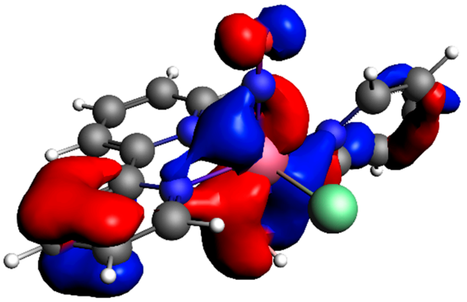 Ru (34), OH (17), NO (7), organic ligands(25)
92	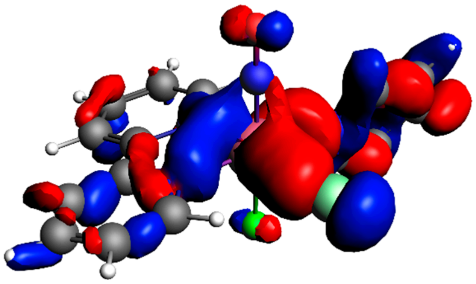 Ru (13), Cl (12), organic ligands (50)	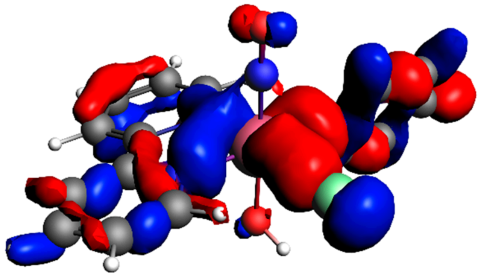 Ru (6), Cl (10), organic ligands (50)
93	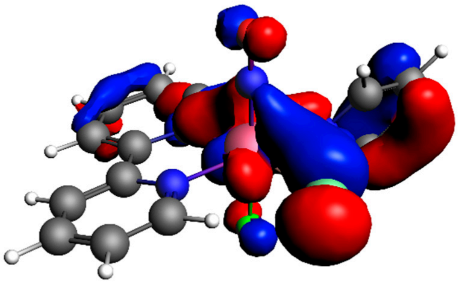 Ru (21), Cl (20), organic ligands (39)	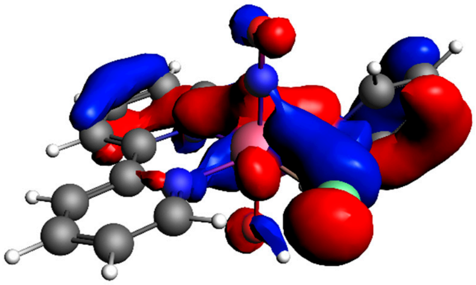 Ru (19), Cl (14), organic ligands (46)
94	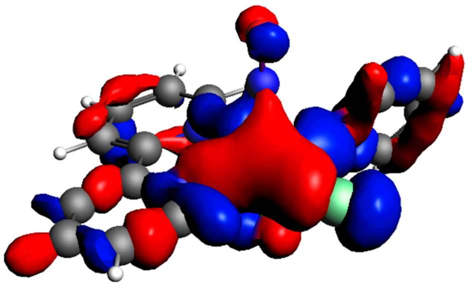 Ru (12), Cl (13), F (5), NO (5), organic ligands (35)	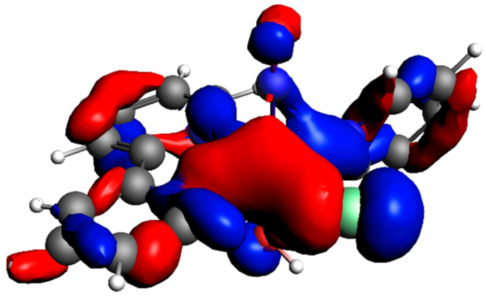 Ru (16), Cl (18), OH (9), NO (7), organic ligands (28)

95	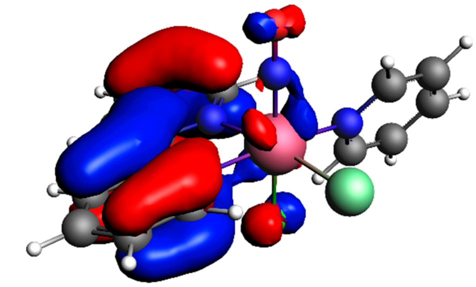<br>Ru (7), Cl (5), F (19), organic ligands (52)	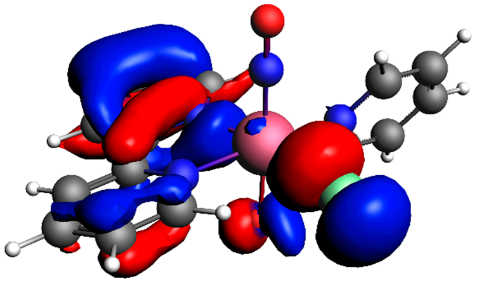<br>Cl (24), OH (4), organic ligands (50)
96	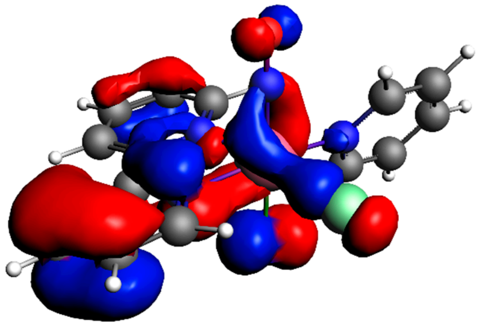<br>organic ligands (78)	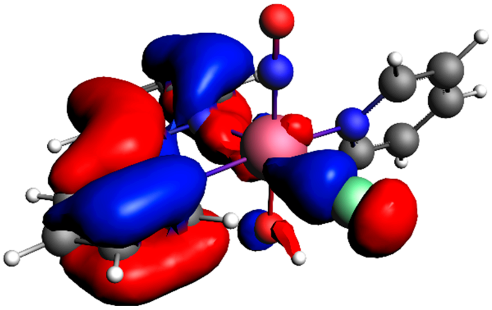<br>Cl (9), organic ligands (73)
97	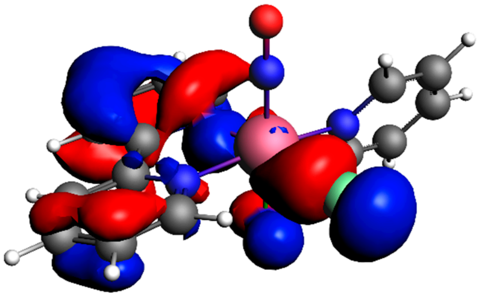<br>Cl (20), F (11), organic ligands (54)	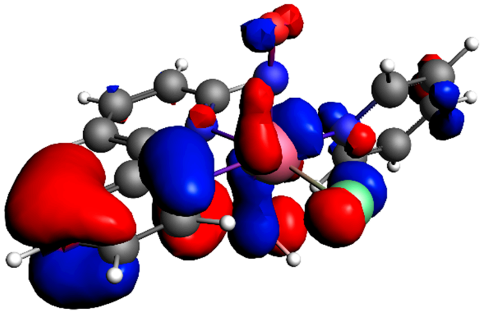<br>Ru (4), Cl (4), OH (8), organic ligands (66)
98	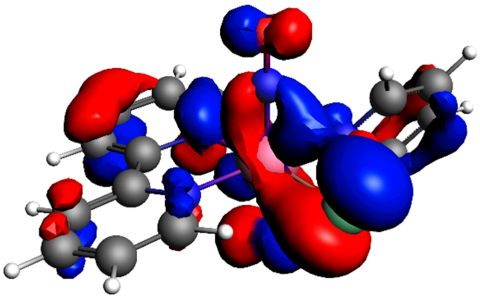<br>Ru (16), Cl (22), F (13), NO(7) organic ligands (22)	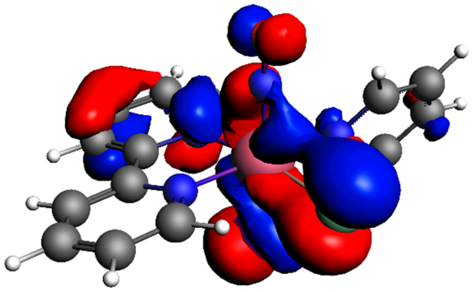<br>Ru (16), Cl (21), OH (26), organic ligands (23)

99	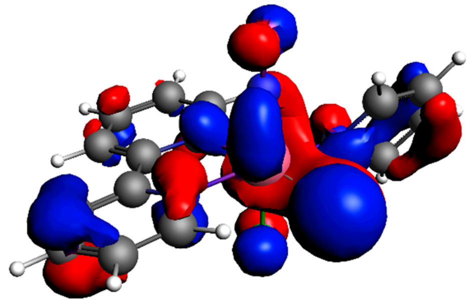 Cl (21); F (8), Ru (17), NO (7), organic ligands (42)	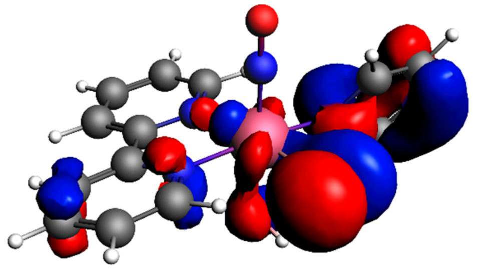 Cl (31), Ru (6), organic ligands (42)
100	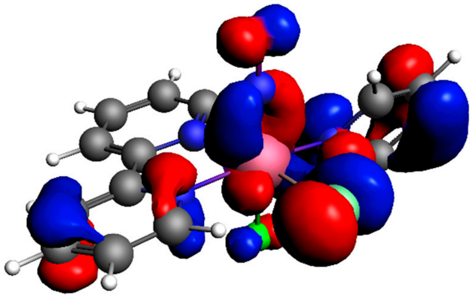 Cl (15), Ru (19), NO (8), organic ligands (42)	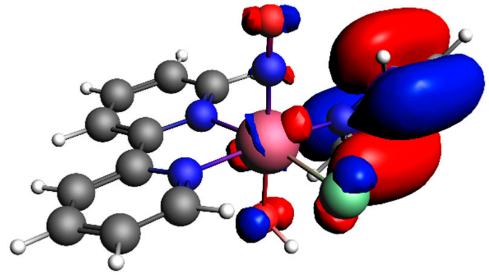 organic ligands (82)
101	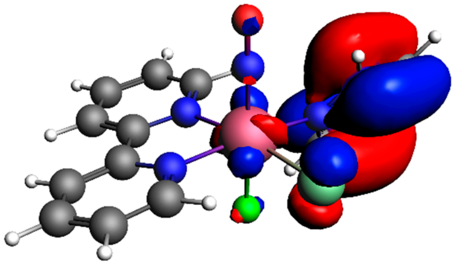 organic ligands (80)	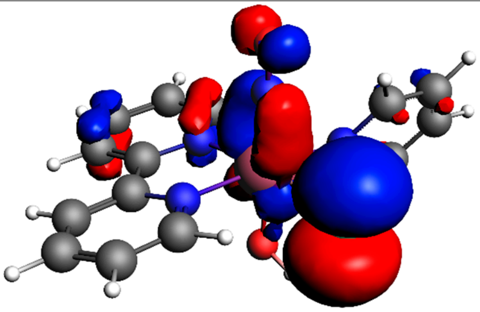 Cl (59), Ru (15), NO (7)
102	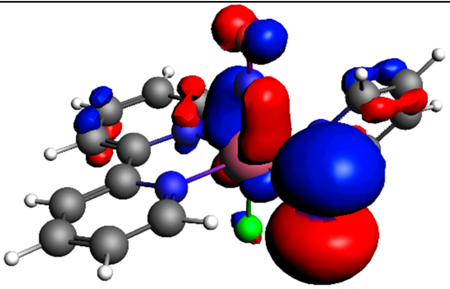 Cl (61), Ru (12), NO (7)	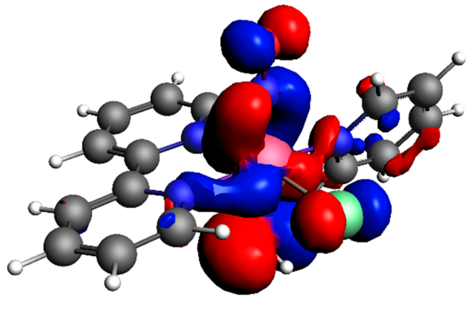 0H (39), Ru (27), NO (12), Cl (6)

103	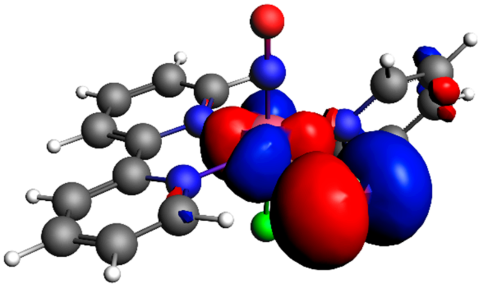 Cl (55), Ru (24)	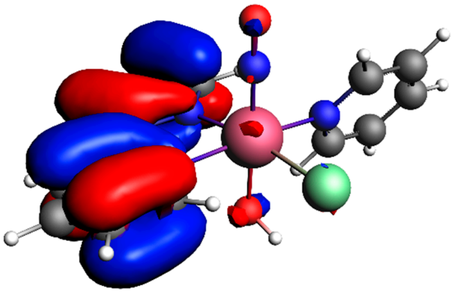 organic ligands (84)
**104 (HOMO)**	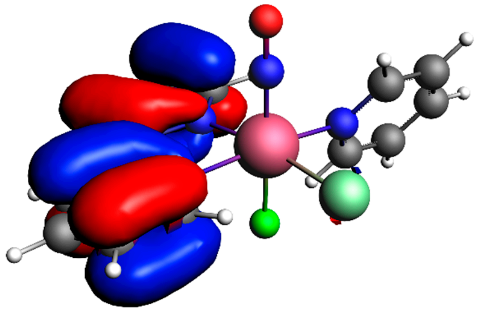 organic ligands (87)	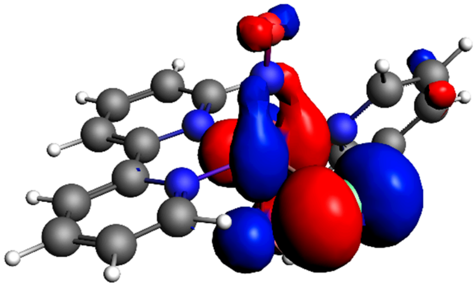 Cl (36), Ru (34), OH (14), NO (3)
**105 (LUMO)**	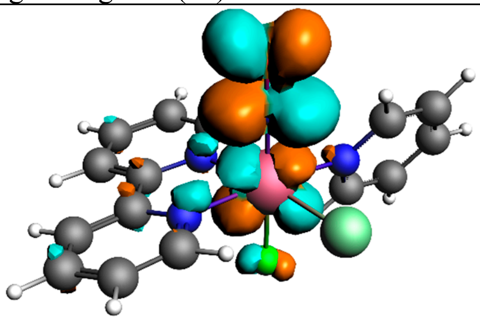 Ru (21), NO (66)	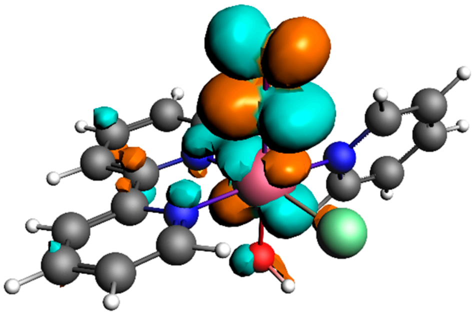 Ru (19), NO (65)
106	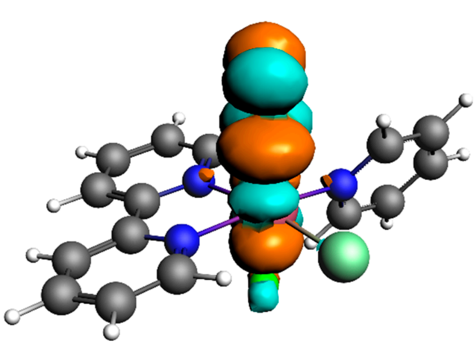 Ru (24), NO (68)	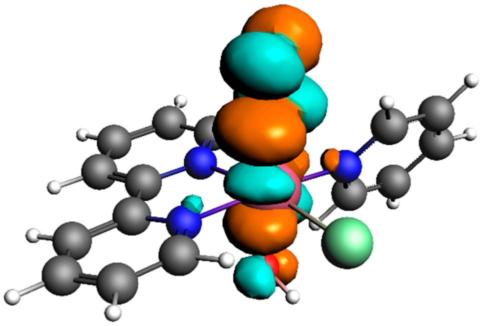 Ru (25), NO (65)

107	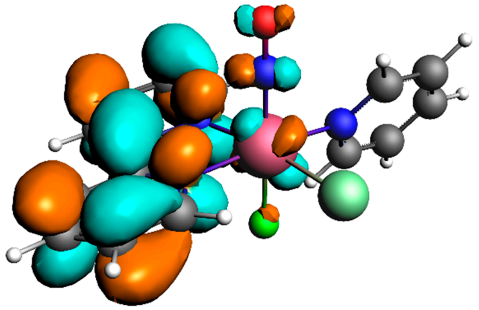 organic ligands (83)	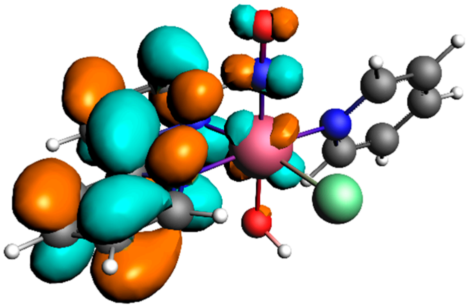 organic ligands (83)
108	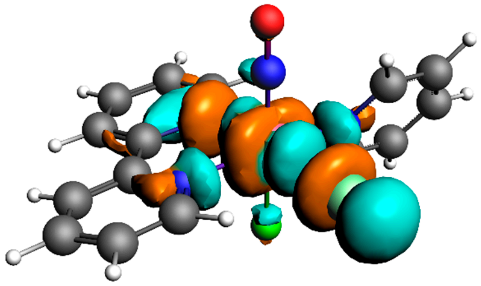 Ru (58), Cl (13), N_{Py} (17)	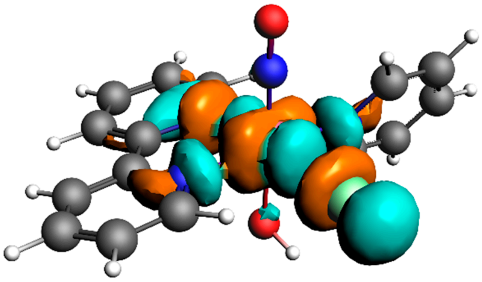 Ru (59), Cl (12), N_{Py} (21)
109	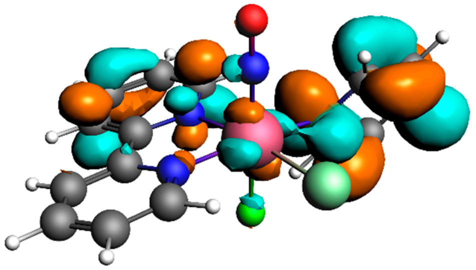 organic ligands (80)	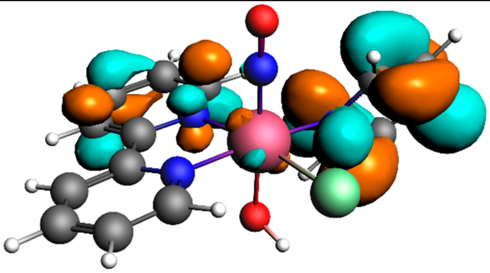 organic ligands (80)
110	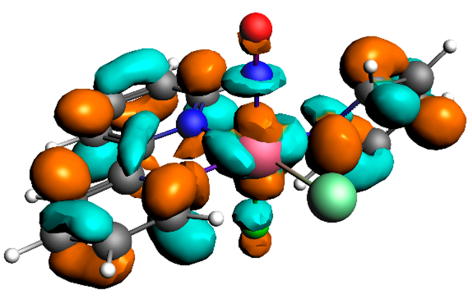 Ru (10), organic ligands (61)	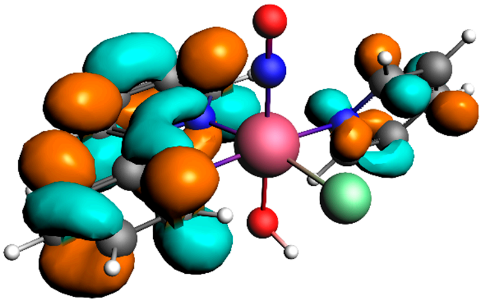 organic ligands (77)
111	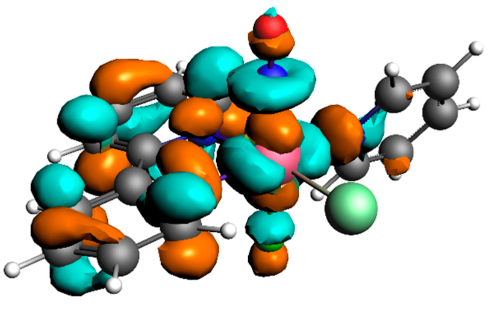 Ru (35), organic ligands (43)	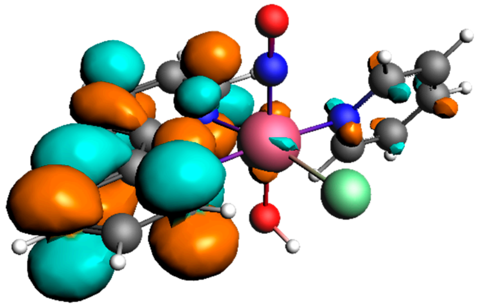 organic ligands (83)
